# From Reactive to Proactive: A QIP for Improving Physical Health Monitoring and Management for Children and Young People with Eating Disorders

**DOI:** 10.1192/bjo.2025.10406

**Published:** 2025-06-20

**Authors:** Sanaa Moledina, Bronwyne Stott

**Affiliations:** Northamptonshire Healthcare NHS Foundation Trust (NHFT), Northampton, United Kingdom

## Abstract

**Aims:** Effective monitoring of children and young people (CYP) with eating disorders (ED) is vital to prevent complications such as malnutrition, electrolyte imbalances, and refeeding syndrome. Assessments at the Northamptonshire Healthcare NHS Foundation Trust Children and Young People Community Eating Disorder Service (NHFT CYPCEDS) & Leicestershire CAMHS Eating Disorder Team (LPT CAMHS EDT) revealed poor coordination between primary care, the clinics, and paediatric hospitals, coupled with limited staff knowledge of physical complications in ED and their management. This contributed to delayed and substandard referrals, high-acuity cases, and increased paediatric admissions. Reliance on GPs, owing to capacity issues, resulted in inconsistent and subpar physical monitoring. Therefore, we aimed to improve physical health monitoring and management for CYP with known ED in Northamptonshire and Leicestershire.

**Methods:** CYPCEDS established a dedicated physical health department, introducing regional clinics in Northamptonshire to improve accessibility. SOPs were developed to streamline physical health management, including refeeding protocols and escalation pathways, while a MEED-based physical health template was integrated into the Trust’s software. A physical health-focused referral letter and the Early Risk Management (ERM) Guide were provided to primary care, explaining ED, its red flags, and key helplines. Staff training workshops were conducted, and innovations like point-of-care machines were implemented. Collaboration with LPT CAMHS supported shared learning. The QIP’s impact was evaluated through audits of paediatric admissions, referrals, and physical health templates, and staff and patient feedback.

**Results:** Electronic physical health template ensured consistent baseline assessments for all new referrals and monitoring during follow-ups. High-acuity referrals were addressed within 24 hours by the Service, while physical health nurses and structured referral pathways reduced the psychiatrists’ workload, improving waitlists and caseload management. Refeeding monitoring improved, with most blood work done in-service using point-of-care technology, reducing reliance on external providers like GPs. Patient feedback highlighted better accessibility and satisfaction, with remote clinics boosting attendance. Staff confidence in managing physical health complications increased, and primary and tertiary teams felt well-supported by joint pathways.

**Conclusion:** The QIP addressed gaps in physical health care for CYP with ED in the region by establishing physical health departments, improving monitoring, standardizing assessments, training staff, optimizing resources, and enabling timely escalation. These improvements led to better patient outcomes, reduced physical acuity, and fewer hospital admissions. Referrals were streamlined, and collaboration between primary, tertiary services, and LPT ensured high-quality care across the region. Future plans include monitoring patient outcomes, creating an SOP repository, and piloting a GP early warning system.

